# Rapid Physiological Trait Measurements in Wine Grape ( *Vitis vinifera* ) Varieties Using the Dynamic Assimilation Technique

**DOI:** 10.1002/pei3.70077

**Published:** 2025-09-12

**Authors:** Leeladarshini Sujeeun, Marney E. Isaac, Kimberley Cathline, Gavin Robertson, Adam R. Martin

**Affiliations:** ^1^ Department of Physical and Environmental Sciences University of Toronto Scarborough Canada; ^2^ Horticultural & Environmental Sciences Innovation Centre Niagara College Canada

## Abstract

Quantifying crop responses to increasing temperatures is critical for predicting the productivity and sustainability of agricultural systems under environmental change. Physiological trait data associated with maximum Rubisco carboxylation (*V*_cmax_) and maximum electron transport (*J*_max_) rates are especially important predictors of crop response to elevated temperatures. However, when generating *V*_cmax_ and *J*_max_ data, steady‐state methods of gas exchange measurements are time‐consuming; thus, non‐steady‐state methods have been developed to obtain these measurements faster, prospectively allowing for trait data collection of considerably more varieties of crops. Globally important and geographically widespread vineyards are of particular interest due to the high economic value and the susceptibility of these managed systems to climate warming, especially in Canada, where the annual rate of warming far exceeds global averages. In this study, we examined the efficacy of the high‐throughput, non‐steady‐state dynamic assimilation technique (DAT) for obtaining *V*_cmax_ and *J*_max_ data from wine grapes. Specifically, we measured *V*_cmax_ and *J*_max_ (alongside leaf nitrogen [N] concentrations and leaf mass per unit area [LMA]) across seven of the world's most common wine grape ( *Vitis vinifera* L.) varieties, namely, Cabernet franc, Cabernet sauvignon, Merlot, Pinot noir, Riesling, Sauvignon blanc, and Viognier. Our results show that *V*_cmax_ and *J*_max_ estimates derived from the DAT were strongly correlated to those obtained through the steady‐state method (*r* ^2^ = 0.748 and 0.908, respectively), and *J*_max_ did not differ significantly between the two methods. Additionally, leaf N explained 43%–46% and 56%–58% of the variation in *V*_cmax_ and *J*_max_, respectively, across both methods. Our results suggest that the DAT represents a viable tool for rapidly estimating intraspecific variation in important physiological traits and allows for increased replication and the inclusion of additional varieties when evaluating the responses of wine grape and other crops to climate warming.

## 1 Introduction

Plant responses to environmental changes can be reliably predicted through the quantification of plant functional traits, including morphological, physiological, or phenological characteristics, which can vary both among species and within species (Díaz and Cabido 1997; Lavorel and Garnier 2002; Westoby and Wright 2006; Funk et al. 2017). One of the most well‐studied approaches to understanding and predicting plant performance in response to environmental changes is the Leaf Economics Spectrum (LES), which includes six core leaf traits—maximum photosynthetic assimilation (*A*_max_), dark respiration rate (*R*_d_), leaf nitrogen (N) and phosphorus (P) concentrations, leaf mass per area (LMA), and leaf lifespan (LL)—that covary with each other across and within plant species (Reich et al. 1999; Wright et al. 2004; Wright et al. 2005). The LES theory posits that plant resource‐uptake and‐use strategies fall along a gradient ranging from resource‐conserving to resource‐acquiring strategies, with resource‐acquiring plants expressing high rates of *A*_max_ and *R*_d_, high leaf N, low LMA, and short LL on the one end and resource‐conserving plants expressing the opposite suite of traits on the other end.

Furthermore, studies have shown that physiological variation along the LES, namely variation in photosynthetic capacity (e.g., *A*_max_), is underpinned by variation in both maximum Rubisco carboxylation (*V*_cmax_) and maximum electron transport rates (*J*_max_) (Xiong and Flexas 2018; Muir et al. 2017; Onoda et al. 2017; Onoda and Wright 2018). Specifically, at low CO_2_ concentrations, photosynthesis is constrained by *V*_cmax_, whereas at high CO_2_ concentrations, photosynthesis is limited by RuBP regeneration, which is largely dependent on *J*_max_ (Sharkey et al. 2007). *V*_cmax_ and *J*_max_ can in turn be affected by leaf N due to the substantial amount of nitrogen invested in Rubisco, which is a crucial component of photosynthesis (Lu et al. 2020; Luo et al. 2021). *V*_cmax_ and *J*_max_ have also been shown to be influenced by LMA (Han et al. 2008; Song et al. 2021), likely due to its impact on nitrogen use efficiency and nitrogen allocation to Rubisco and thylakoids (Hassiotou et al. 2010).

Most research on LES trait variation in natural or unmanaged ecosystems has focused on characterizing interspecific differences in trait expression. This focus is largely based on the assumption that trait variation within species is smaller than the variation that exists among species (McGill et al. 2006), with some studies showing that intraspecific variation does not alter a species' trait‐based rank within a community (Garnier et al. 2001; Hulshof and Swenson 2010; Mudrák et al. 2019). However, recent studies have shown that intraspecific trait variation can account for 25%–30% of the total LES trait variation within plant communities (Fajardo and Siefert 2016; Fajardo and Siefert 2018; Siefert et al. 2015; Albert et al. 2010). Trait differences among individuals of the same plant species can be driven by environmental factors, including temperature and precipitation regimes (Siefert et al. 2015; Albert et al. 2010) and can in turn influence population‐scale, community‐scale, and ecosystem‐scale processes (reviewed by Westerband et al. 2021).

Intraspecific trait variation has also been shown to contribute to differences in several agroecosystem services and functions, such as productivity (Bouman and van Laar 2006; Gagliardi et al. 2015), light interception (Milla et al. 2014), and litter decomposition and nutrient cycling (He et al. 2012; García‐Palacios et al. 2013). In managed environments, including agricultural systems—the focus of our work here—quantifying trait variation within crop species is especially important, as the traits of only a few species or varieties of a given crop drive ecosystem functioning (Martin and Isaac 2015; Milla et al. 2015) and because different varieties of the same crop may differ in their responses to environmental changes (Prasad et al. 2006; Glaubitz et al. 2013; Gallo et al. 2021).

Quantifying trait variation in wine grape is of particular interest as it is one of the most widely cultivated and economically important crops. For example, in 2023, the global wine industry generated a total revenue of over $330 billion USD (Statista 2024). In Canada alone, wine production contributes ~$9–11.5 billion USD to the national economy annually (Hewer and Gough 2020). However, over the past few decades, the global vineyard area has been declining consistently (International Organisation of Vine and Wine 2024) due to drastic climatic changes affecting wine grape yield and quality, including elevated temperatures during the growing season (e.g., Liles and Verdon‐Kidd 2020), higher frequency of extreme temperature events (e.g., White et al. 2006), and lower water availability (e.g., Santillán et al. 2019). These environmental changes have been shown to affect wine grape physiology, phenology, and growth, with a direct impact on the productivity of vineyards (van Leeuwen et al. 2024). The climatic effects on wine grape production may be even more pronounced in Canada, where the annual average temperature has increased by 2.0°C from 1948 to 2023, approximately twice the rate as the global average temperature (Environment and Climate Change Canada 2024). Therefore, given the high economic importance of wine grape production, there is an urgent need to quantify and predict the responses of wine grapes to environmental changes in vineyards.

LES traits such as *A*_max_ and the related key parameters of photosynthetic capacity, *V*_cmax_ and *J*_max_, are highly temperature‐dependent (Fan et al. 2011; Lu et al. 2022) and are thus essential for quantifying wine grape performance in response to environmental changes (e.g., Greer and Weedon 2012; Gallo et al. 2021). However, the collection of data on these traits is very labor‐intensive and time‐consuming. For example, *V*_cmax_ and *J*_max_ are typically derived from steady‐state photosynthetic CO_2_ response (*A*‐*C*_i_) curves obtained through leaf‐level CO_2_ gas exchange measurements in the field using portable infrared gas analyzers, where each curve can take up to 40 min to generate (Fan et al. 2011; Muir et al. 2017; Tejera‐Nieves et al. 2024). Given these constraints, sampling is commonly limited to few individuals and/or varieties of wine grapes and other crops. Consequently, high‐throughput phenotyping (HTP) methods such as the dynamic assimilation technique (DAT) are being developed to allow for more rapid gas exchange measurements (Saathoff and Welles 2021; Tejera‐Nieves et al. 2024).

Traditional methods for gas exchange measurements have long required steady‐state conditions to generate high‐accuracy data. However, each data point on a steady‐state *A*‐*C*_i_ curve takes 1–3 min, and a full *A*‐*C*_i_ curve typically requires 25–40 min in order to obtain a sufficient number of points for *V*_cmax_ and *J*_max_ estimations (Saathoff and Welles 2021; Tejera‐Nieves et al. 2024). In addition, long measurement times at low CO_2_ concentrations may alter the physiological state of the leaf through the deactivation of Rubisco, which can result in lower *V*_cmax_ estimates than would be representative under ambient conditions (Caemmerer and Edmondson 1986; Tejera‐Nieves et al. 2024). To address these constraints, Saathoff and Welles (2021) recently introduced the DAT to obtain CO_2_ response curves under non‐steady‐state conditions by continuously ramping CO_2_ concentrations during measurements.

Unlike traditional steady‐state gas exchange measurements, the DAT does not require stable CO_2_ concentrations in the leaf chamber before assimilation can be measured, resulting in faster *A*‐*C*_i_ curves. The few studies that have compared photosynthetic parameters using the DAT vs. steady‐state methods on crop species have shown that *V*_cmax_ and *J*_max_ data were nearly identical across both methods (Saathoff and Welles 2021; Tejera‐Nieves et al. 2024). Despite these promising results, to date, no studies have attempted to determine whether the DAT is able to quantify differences in *V*_cmax_ and *J*_max_ across crop varieties without compromising data accuracy or precision. This is a critical research gap that needs to be addressed, given that intraspecific trait variation plays an essential role in various agroecological services, as mentioned above, and that such high‐throughput methods are rarely tested across different varieties of the same species.

In this study, we assess for the first time whether or not the DAT can be used to quantify intraspecific variation in *V*_cmax_ and *J*_max_ across some of the most widely cultivated wine grape varieties. Specifically, we compare estimates of *V*_cmax_ and *J*_max_ derived from *A*‐*C*_i_ curves under steady‐state conditions using the steady‐state method and under non‐steady‐state conditions using the DAT. Here, we address the following research questions: 1) Are there significant differences in *V*_cmax_ and *J*_max_ values between wine grape varieties? 2) If so, can the non‐steady‐state DAT reliably and rapidly quantify differences in *V*_cmax_ and *J*_max_ among wine grape varieties? 3) How well do values of *V*_cmax_ and *J*_max_ derived from non‐steady‐state gas exchange methods match those obtained from steady‐state gas exchange data? And finally, 4) are *V*_cmax_ and *J*_max_ values from the DAT and steady‐state method correlated with LES traits?

## 2 Materials and Methods

### 2.1 Study Site and Sample Collection

This study was conducted at the Niagara College Teaching Vineyard, Niagara‐on‐the‐Lake, Ontario, Canada (43.1522° N, 79.1652° W). This 16.2 ha operational vineyard, located in the Niagara Peninsula Appellation in Southern Ontario, Canada, is not irrigated and is characterized by imperfectly drained silty clay over clay loam, mixed with poorly drained lacustrine heavy clay. Based on climate data at a 1‐km^2^ resolution (Fick and Hijmans 2017), the annual average temperature at the vineyard is 8.6°C, with a mean annual precipitation of 949 mm. The site is uniformly tilled and sprayed with calcium nitrate and/or muriate of potash and/or sulfate of potash magnesium (K‐Mag; 22–10.8‐22) in mid‐June. Additionally, liquid calcium (8–0–0‐10) is sprayed on the leaves early in the growing season (Macklin et al. 2022; Martin et al. 2022).

We obtained trait data for seven of the most common wine grape varieties grown at the site—Cabernet franc (clone 314), Cabernet sauvignon (clones 29 and 412), Merlot (clones 181 and 348), Pinot noir (clones 89 and 828), Riesling (clone 171), Sauvignon blanc (clone 906), and Viognier (clone 642). All the vines used in this study were grafted to rootstock 3309 C, with the exception of Viognier (which was grafted to rootstock 101–14 MgT), and they were trained using a 2‐arm flat vertical shoot position system. Cabernet franc (clone 314), Cabernet sauvignon (clones 29 and 412), and Merlot (clones 181 and 348) were planted in 2019, while Pinot noir (clones 89 and 828), Sauvignon blanc (clone 906), and Viognier (clone 642) were planted in 2020. All trait data were collected from June 10–19, 2024 (between 8:00 and 14:00), when the vines were in the fruit setting (E‐L 27) stage. We sampled *n* = 3 vines (one per planting row) for the Cabernet franc, Riesling, Sauvignon blanc, and Viognier varieties and six vines (one per planting row) for the Cabernet sauvignon, Merlot, and Pinot noir varieties, for a total of *n* = 30 individual vines. One leaf from each vine was selected from the uppermost section of the plant for trait data collection, ensuring that all leaves were fully expanded and sun‐exposed, newly developed, and free of any pest, pathogen, or mechanical damage (Perez‐Harguindeguy et al. 2016). All sampled leaves were taken from the northwest side of the vineyard and were positioned approximately 5–7 nodes below the apical bud on the corresponding shoot.

### 2.2 A‐Ci Curve Data Collection

The trait data that were collected in our study include *V*_cmax_, *J*_max_, leaf N concentration, and LMA. We obtained *A‐C*_*i*_ curve data (*V*_cmax_ and *J*_max_) in the field using an LI‐6800 portable photosynthesis system affixed with a 6800–03 large light source (Licor Biosciences, Lincoln, Nebraska, USA), using both the DAT and steady‐state method. Specifically, two *A‐C*_*i*_ curves were performed on each leaf, with one curve for each method (Saathoff and Welles 2021; Gregory et al. 2023; McClain and Sharkey 2023). For each curve obtained using the DAT, area‐based CO_2_ assimilation rates (*A*_area_; *μ*mol CO_2_ m^−2^ s^−1^) were logged every 2 s across continuously ramping CO_2_ concentrations, with a ramp rate of 100 *μ*mol mol^−1^ min^−1^ (based on the recommendations by Stinziano et al. 2019 and McClain and Sharkey 2023), starting at 50 *μ*mol mol^−1^ and ending at 1500 *μ*mol mol^−1^. This low ramp rate was chosen to avoid acute triose phosphate utilization (TPU) limitation, commonly indicated by oscillations in *A*_net_ that occur at high CO_2_ concentrations under conditions of rapid change in *C*_i_ (McClain and Sharkey 2023). A split down‐up approach was used for steady‐state *A‐C*_*i*_ curves since this is one of the most commonly used protocols for gas exchange measurements (e.g., Greer and Weedon 2012; Moualeu‐Ngangue et al. 2017; Coursolle et al. 2019; Cavanagh et al. 2022; Gallo et al. 2021), making it highly relevant for comparison against the DAT. Specifically, steady‐state *A‐C*_*i*_ curves were obtained using the following setpoints: 400, 300, 200, 100, 50, 0, 400, 400, 600, 800, 1000, 1200, and 1500 *μ*mol mol^−1^. Otherwise, leaf chamber conditions were maintained as follows: photosynthetic photon flux density (PPDF) of 1500 *μ*mol m^−2^ s^−1^ of photosynthetically active radiation (with irradiance peaks in blue [453 nm], green [523 nm], and red [660 nm] wavebands), relative humidity of 60%, leaf temperature of 25°C, and leaf vapor pressure deficit of 1.7 kPa. In addition, the CO_2_ and H_2_O sensors were readjusted after three to four *A‐C*_*i*_ measurements using the range match function. All leaves were allowed to acclimate to chamber conditions for ~10 min between all *A‐C*_*i*_ curves. All paired steady‐state/DAT *A‐C*_*i*_ curves were obtained within 24 h of each other, in a randomized order. Each DAT *A‐C*_*i*_ curve required approximately 15 min, whereas a steady‐state *A‐C*_*i*_ curve required ~26 min, including a 60–120 s acclimation period (Saathoff and Welles 2021).

After obtaining all *A*‐C_i_ data in the field, we collected and transported all sampled leaves to the University of Toronto Scarborough, Canada for the determination of LMA and leaf N concentration. Following the removal of the petioles, the area of fresh leaves was measured using an LI‐3100C leaf area meter (Licor Bioscience, Lincoln, Nebraska, USA) and then dried for 48 h until a constant mass was reached. Using the weight of the dried leaves, LMA was calculated as leaf dry mass/leaf area. Finally, the dried leaves were ground to a fine and homogeneous powder (< 0.5 mm) using a MM400 Retsch ball mill (Retsch Ltd., Hann, Germany), and ~0.15 g of each ground sample was analyzed to obtain leaf N concentration using a LECO CN 628 elemental analyzer (LECO Instruments, Ontario, Canada).

### 2.3 Statistical Analysis

**FIGURE 1:**
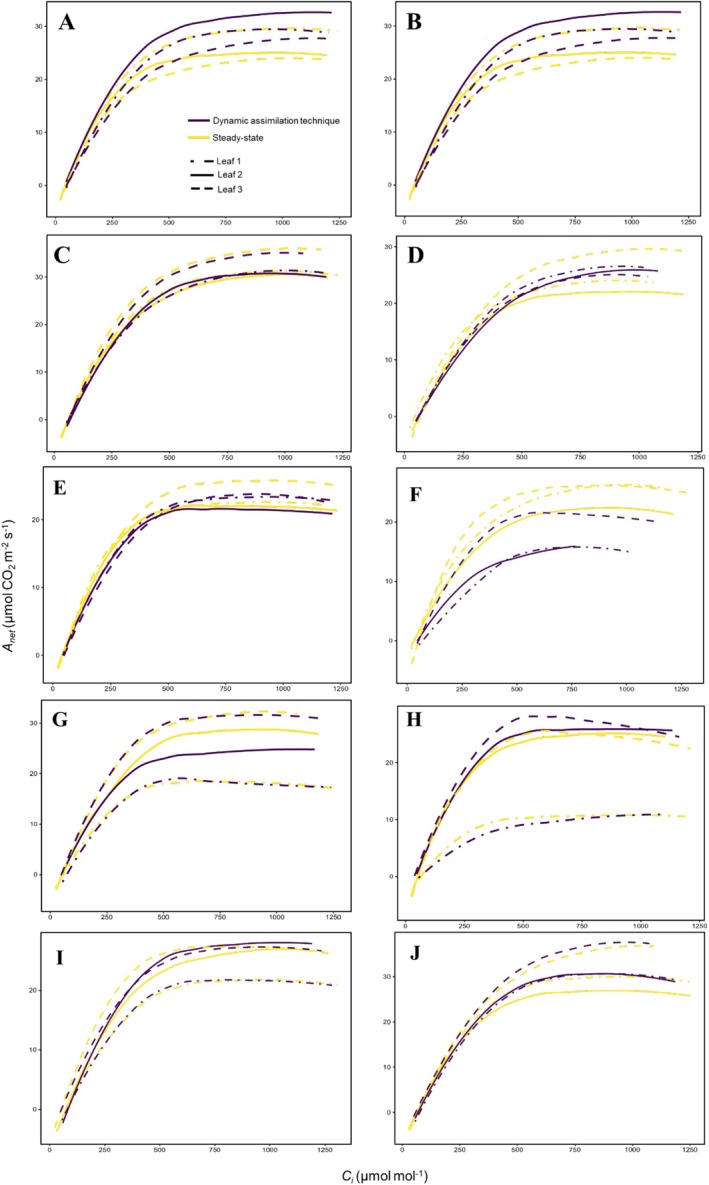
Net CO_2_ assimilation (*A*_net_) response to intercellular CO_2_ concentration (*C*_i_) in 10 wine grape clones (A: Cabernet franc clone 314; B: Cabernet sauvignon clone 29; C: Cabernet sauvignon clone 412; D: Merlot clone 181; E: Merlot clone 348: F: Pinot noir clone 828; G: Pinot noir clone 89; H: Riesling clone 171; I: Sauvignon blanc clone 906; J: Viognier clone 642) using the dynamic assimilation technique (DAT) and steady‐state method. Each panel represents paired *A*‐*C*_i_ curves derived from the DAT and steady‐state method for *n* = 3 leaves.

All statistical analysis and data visualization were performed using R statistical software v. 4.2.1 (R Foundations for Statistical Computing, Vienna, Austria). The rates of *V*_cmax_ and *J*_max_, along with their standard errors, were estimated by fitting the Farquhar, von Caemmerer and Berry (FvCB) model to each *A*‐*C*_i_ curve using the “fitaci” function in the “plantecophys” R package (Duursma 2015). Since the physiology of TPU limitation is interpreted differently under dynamic and steady state conditions, and because our DAT were designed to avoid acute TPU limitation (McClain and Sharkey 2023), we did not consider TPU in either analysis. The FvCB models were fit to the *A*‐*C*_i_ curves using nonlinear least square regression (Duursma 2015); *V*_cmax_ and *J*_max_ were corrected to 25°C and were considered to be apparent given that mesophyll conductance was assumed to be infinite. Paired *A*‐*C*_i_ curves derived from the DAT and steady‐state method for each clone are shown in Figure 1.

Using the extracted *V*_cmax_ and *J*_max_ mean and standard error values, we then conducted a paired *t*‐test to test for statistically significant differences in *V*_cmax_ and *J*_max_ between the DAT and steady‐state methods. Then, we performed Deming regression analyses (where *n* = 30 leaves) to determine whether *V*_cmax_ and *J*_max_ derived from the DAT predict *V*_cmax_ and *J*_max_ derived from the steady‐state method. These regression models were supplemented with a linear hypothesis test, implemented using the “car” R package (Fox et al. 2007), which compared these regression models to a 1:1 relationship where *V*_cmax_ and *J*_max_ obtained using the DAT perfectly correspond to *V*_cmax_ and *J*_max_ derived from the steady‐state method. One‐way ANOVA followed by post hoc Tukey HSD tests was used to test for significant differences in *V*_cmax_ and *J*_max_ derived from the steady‐state method vs. the DAT. We also used Spearman rank correlation tests to evaluate the degree to which the *A*‐C_i_ curve methodology affects the rankings of grapevine varieties based on *V*_cmax_ and *J*_max_. Finally, we performed linear regression models (where *n* = 30 leaves) to determine whether *V*_cmax_ and *J*_max_ were correlated with leaf N concentration and LMA, and whether the strength of these relationships differed across methodologies.

## 3 Results

**FIGURE 2:**
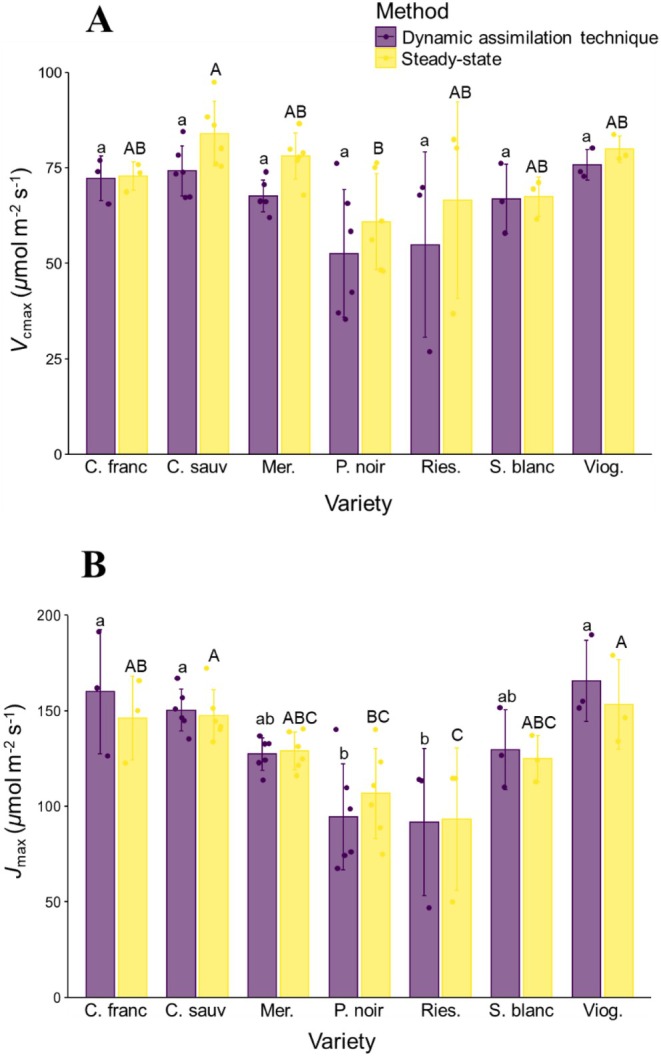
Variety‐level maximum Rubisco carboxylation (*V*_cmax_) (A) and maximum electron transport (*J*_max_) (B) rates for seven wine grape varieties through high‐throughput (dynamic assimilation technique) and traditional (steady‐state) methods. Individual points correspond to individual leaf‐level observations of *V*_cmax_ (A) and *J*_max_ (B), while bars correspond to variety‐level mean values (±1 SE). Significant differences between varieties with the DAT and steady‐state method at *p* < 0.05 (Tukey HSD) are shown by different lowercase and uppercase letters, respectively.

Based on our paired *t*‐tests, we uncovered statistically significant differences in mean *V*_cmax_ (*t* = 5.95, d.f. = 29, *p* < 0.001) but not in mean *J*_max_ (*t* = −0.35, d.f. = 29, *p* = 0.73) across methods. Specifically, across wine grape varieties, *V*_cmax_ values measured using the steady‐state method averaged 73.3 ± 12.9 (s.d.) *μ*mol m^−2^ s^−1^, ranging from 60.9 ± 5.1 *μ*mol m^−2^ s^−1^ (s.e.) in Pinot noir to 84.0 ± 3.4 *μ*mol m^−2−1^s in Cabernet sauvignon (Figure 2A). Mean *V*_cmax_ based on data derived from the DAT was 65.9 ± 13.7 (s.d.) *μ*mol m^−2^ s^−1^, ranging from 52.6 ± 6.8 *μ*mol m^−2^ s^−1^ in Pinot noir to 75.7 ± 2.3 *μ*mol m^−2^ s^−^1 in Viognier (Figure 2A). Average *J*_max_ obtained from steady‐state curves was 128.4 ± 26.8 (s.d.) *μ*mol m^−2^ s^−1^, with a range of 93.2 ± 21.6 *μ*mol m^−2^ s^−1^ in Riesling to 153.3 ± 13.5 *μ*mol m^−2−1^s in Viognier (Figure 2B). Comparatively, mean *J*_max_ based on the DAT was 129.1 ± 33.5 *μ*mol m^−2^ s^−1^, ranging from 91.6 ± 22.3 *μ*mol m^−2^ s^−1^ in Riesling to 165.5 ± 12.2 *μ*mol m^−2−1^s in Viognier (Figure 2B).

**FIGURE 3:**
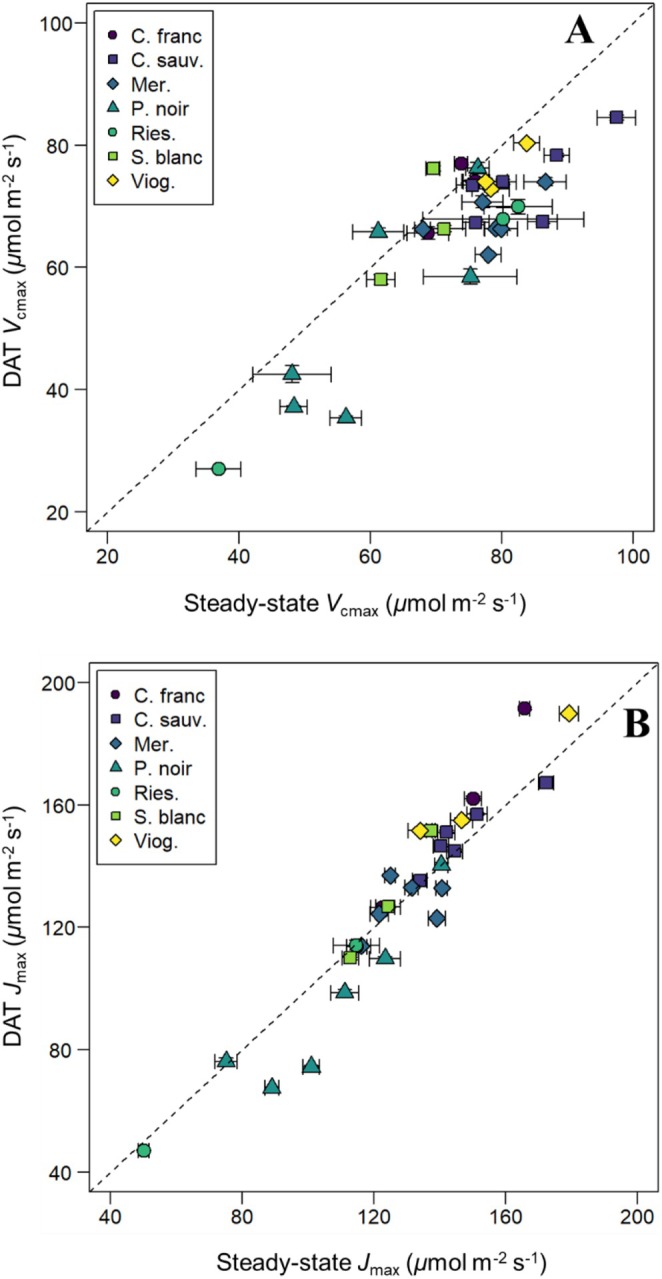
Maximum Rubisco carboxylation (*V*_cmax_) (A) and maximum electron transport (*J*_max_) (B) rates of leaves from seven grapevine varieties estimated through high‐throughput (dynamic assimilation technique) and traditional (steady‐state) methods. Panels A and B display estimates of *V*_cmax_ and *J*_max_ derived through both methods measured on the same leaf of individual vines, respectively. Means are plotted ±1 SE. Each variety is represented by a unique combination of color and shape for clarity. The dashed line represents 1:1 relationships.

Deming regression models indicated that variety‐level mean *V*_cmax_ values derived from the DAT were strongly positively correlated with those obtained using the steady‐state method. Specifically, DAT data explained 75.7% of the variation in *V*_cmax_ values (Deming regression model *p* < 0.001, intercept = 11.4 ± 6.4, slope = 0.94 ± 0.09) (Figure 3A). However, this relationship did differ statistically from a 1:1 relationship as per a linear hypothesis test (*F* = 29.6, *p* < 0.001). Mean *J*_max_ values derived from the DAT were also strongly and positively associated with those obtained using the steady‐state method, such that DAT values explained 91.1% of the variation in steady‐state *J*_max_ values (Deming regression model *p* < 0.001, intercept = 26.2 ± 10.1, slope = 0.79 ± 0.07) (Figure 3B), and this relationship did not differ statistically from a 1:1 relationship (linear hypothesis test *F* = 1.12, *p* = 0.299).

**FIGURE 4:**
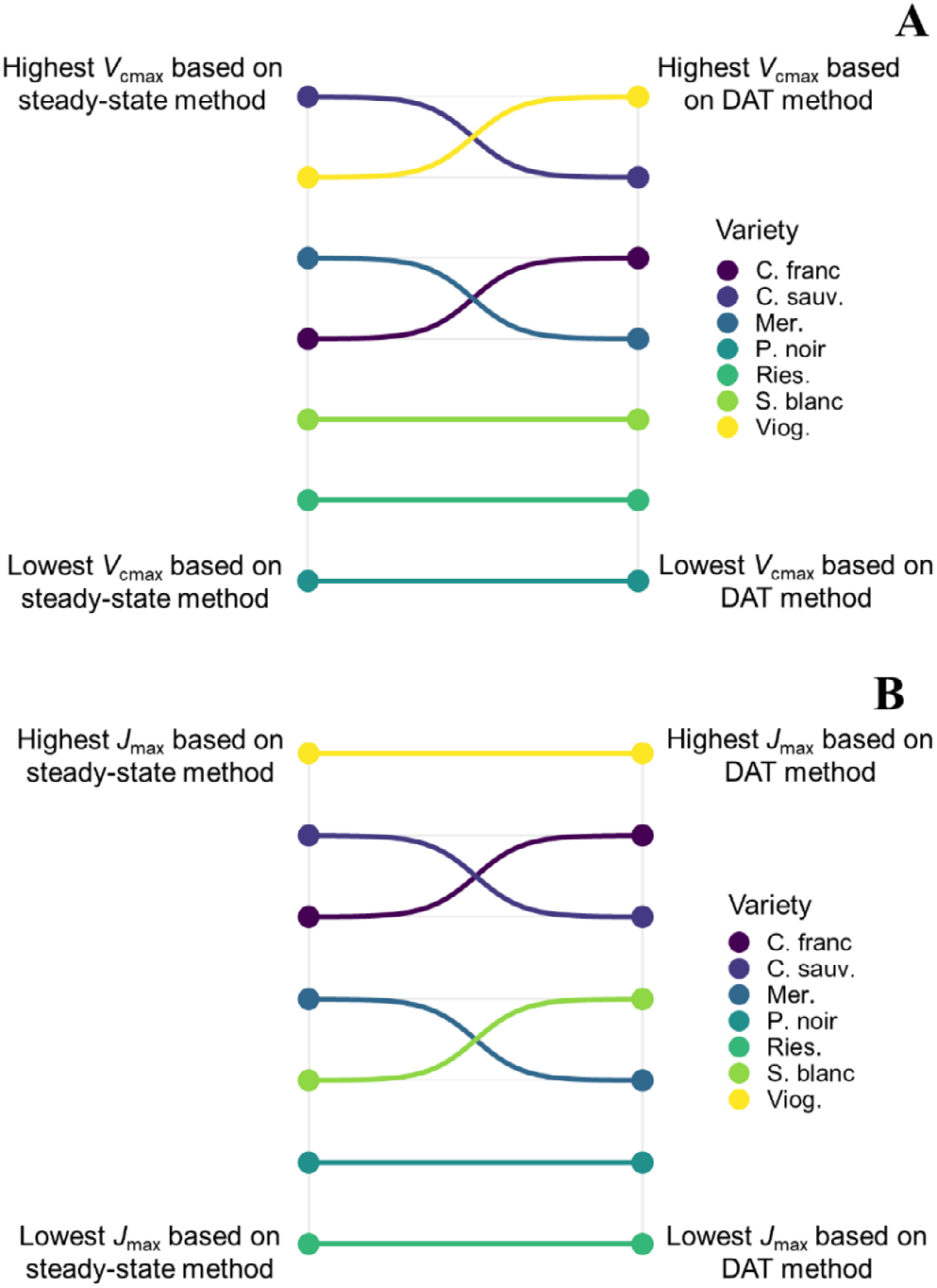
Average values of maximum Rubisco carboxylation (*V*_cmax_) (A) and maximum electron transport (*J*_max_) (B) rates of seven wine grape varieties estimated through high‐throughput (dynamic assimilation technique) and traditional (steady‐state) methods. Panels A and B represent rank shifts in *V*_cmax_ (A) and *J*_max_ (B) for varieties when estimated using both methods. Ordering of the left‐hand side points denotes *V*_cmax_ (A) and *J*_max_ (B) rankings based on the traditional (steady‐state) method, while ordering of the right‐hand side points denotes *V*_cmax_ (A) and *J*_max_ (B) rankings based on the high‐throughput (dynamic assimilation technique) method.

Significant differences between wine grape varieties were detected for both *V*_cmax_ and *J*_max_ values, with these results being similar across both methods (Figure 2). Specifically, with regard to *V*_cmax_, the steady‐state method showed significant differences between Pinot noir and Sauvignon blanc (*p* < 0.05), while the DAT showed an almost significant difference for the same varieties (*p* = 0.053). Similarly, significant differences in *V*_cmax_ observed between varieties were almost identical between both methods. The ranking of wine grape varieties based on their mean *V*_cmax_ and *J*_max_ values also did not differ significantly across methodology (Spearman's *ρ* = 0.67, *p* < 0.001 and Spearman's *ρ* = 0.93, *p* < 0.001, respectively). In particular, the ranks of Pinot noir, Riesling, and Sauvignon blanc based on *V*_cmax_ were most robust toward methodology (Figure 4A), while Pinot noir, Riesling, and Viognier had the same rank based on *J*_max_ across both methods (Figure 4B). In addition, while the ranking of certain varieties changed across methods (e.g., Cabernet franc), all rank shifts were limited to one position for both *V*_cmax_ and *J*_max_ (Figure 4B).

**FIGURE 5:**
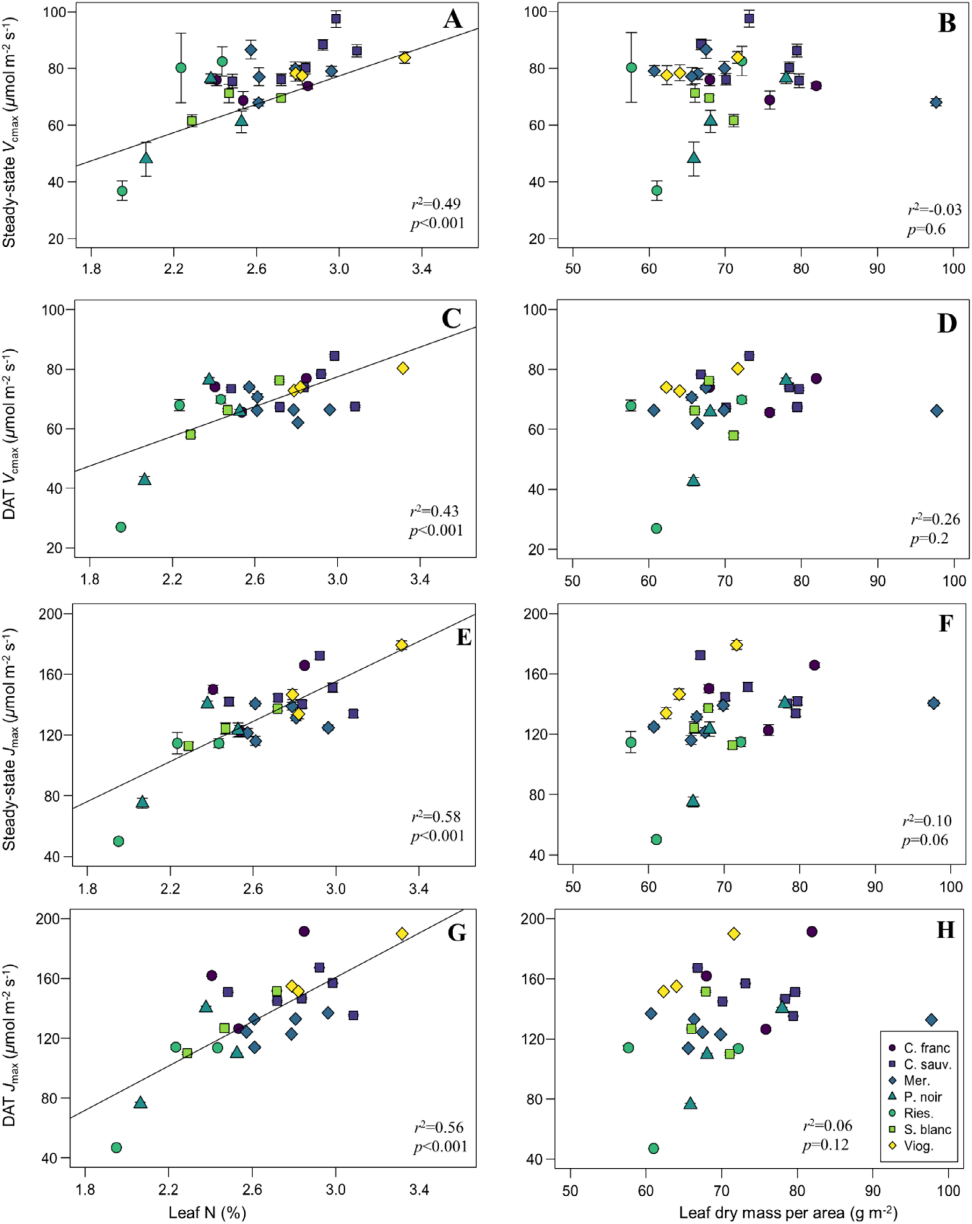
Maximum Rubisco carboxylation (*V*_cmax_) (A–D) and maximum electron transport (*J*_max_) (E–H) rates of seven grapevine varieties estimated through the steady‐state method (A, B and E, F, respectively) and dynamic assimilation technique (C, D and G, H, respectively) in relation to leaf N concentration (A, C, E, and G) and leaf dry mass per area (B, D, F, and H). Means are plotted ±1 SE. Each variety is represented by a unique combination of color and shape for clarity. Solid lines represent significant results (*p* < 0.05).

Finally, linear regression models indicated that inferences surrounding relationships between LES traits and *V_cmax_/J*_max_ were robust toward *A*‐*C*_i_ curve methodology. Specifically, we found strong correlations between mean *V*_cmax_ and *J*_max_ values and leaf N concentration (Figure 5): trends that were robust across both the steady‐state method (*r* ^2^ = 0.49, *p* < 0.001 and *r* ^2^ = 0.58, *p* < 0.001, respectively) and the DAT‐based data (*r* ^2^ = 0.43, *p* < 0.001 and *r* ^2^ = 0.56, *p* < 0.001, respectively). However, correlations between LMA and *V*_cmax_ or *J*_max_ were not significant when analyzing data from the steady‐state method (*r* ^2^ = −0.03, *p* = 0.6 and *r* ^2^ = 0.10, *p* = 0.06, respectively) and the DAT (*r* ^2^ = 0.26, *p* = 0.2 and *r* ^2^ = 0.06, *p* = 0.12, respectively).

## 4 Discussion

Our study is among the first to employ non‐steady‐state gas exchange techniques for evaluating intraspecific variation in crop leaf traits. Specifically, in our study, the DAT of Saathoff and Welles (2021), as employed by a number of studies (Gregory et al. 2023; McClain and Sharkey 2023), returns variety‐level mean *V*_cmax_ values ranging from 53 to 84 *μ*mol m^−2^ s^−1^ and mean *J*_max_ ranging from 92 to 166 *μ*mol m^−2^ s^−1^. Consistent with expectations that domestication has enhanced resource acquisition traits in crops, our values fall above average *V*_cmax_ and *J*_max_ values (measured at 25°C) reported for a wide range of plant species in multiple meta‐analyses (Wullschleger 1993; Leuning 2002; Kattge and Knorr 2007; Walker et al. 2014). Our *J*_max_ and *V*_cmax_ values reported here from both techniques also fall within the range of *J*_max_ and *V*_cmax_ reported for wine grapes and indicate that certain varieties (namely, Viognier and Sauvignon blanc) express high resource‐acquiring values of these traits.

For example, studies on wine grape photosynthetic responses to a multitude of environmental factors, including temperature, drought, and light, report *J*_max_ and *V*_cmax_ (both at 25°C) ranging from ~30 to 230 *μ*mol m^−2^ s^−1^ and ~35 to 85 *μ*mol m^−2^ s^−1^, respectively (de Souza et al. 2005; Greer and Weedon 2013; Li et al. 2017; Greer 2019; Greer 2020). Our analysis then demonstrates the contributions that non‐steady‐state photosynthetic response curves present for better understanding the extent of intraspecific trait variation in crops and other plant species. Indeed, pairing these methods with other high‐throughput techniques, such as trait estimation through reflectance spectroscopy (Burnett et al. 2021; Cui et al. 2025) presents an intriguing prospect for vastly expanding intraspecific trait quantification.

Overall, the DAT appears an efficient approach for obtaining *A*‐*C*_i_ curves and estimating physiological traits such as *V*_cmax_ and *J*_max_ without compromising the accuracy of the data, consistent with the findings of Saathoff and Welles (2021) and Tejera‐Nieves et al. (2024). Estimations of *J*_max_ using the DAT were almost identical to those derived from the traditional steady‐state method, but *V*_cmax_ estimates were slightly lower when derived from the DAT compared to the steady‐state method (Figures 1, 2, 3). Nonetheless, mean *V*_cmax_ using the DAT was only ~10% lower than the average *V*_cmax_ value obtained from the steady‐state method and demonstrates the potential of this technique for measuring physiological traits among different species and across varieties of the same species. The DAT reduced the measurement time of *A*‐*C*_i_ curves by almost half and can potentially be performed even faster by increasing the CO_2_ ramp rate. For example, Saathoff and Welles (2021) obtained similar *V*_cmax_ and *J*_max_ estimates for sunflower plants grown under greenhouse conditions across ramp rates ranging from 100 to 400 *μ*mol mol^−1^ min^−1^, with *A*‐*C*_i_ curves taking only 5–10 min. However, certain species or varieties of crops may require lower ramp rates and thus longer measurement times to prevent large oscillations during gas exchange measurements (McClain and Sharkey 2023; Tejera‐Nieves et al. 2024).

Our findings also indicate that both *V*_cmax_ and *J*_max_ differ significantly among wine grape varieties, likely driven by differences in leaf N concentrations as a proxy for photosynthetic enzymes (Lu et al. 2020; Luo et al. 2021). Indeed, *V*_cmax_ and *J*_max_ were strongly correlated to leaf N concentration—a trend observed using both the steady‐state method and DAT—which explained 43%–46% and 56%–58% of the variation in these traits, respectively. Positive correlations between leaf N concentration and physiological traits such as *V*_cmax_ and *J*_max_ are likely attributable to N allocation toward chlorophyll–protein complexes, as well as thylakoid proteins and enzymes involved in photosynthesis, particularly Rubisco (Evans and Seemann 1989; Rogers et al. 2017; Vårhammar et al. 2015; Lu et al. 2020; Medlyn et al. 1999). As such, plants with higher leaf N concentrations—indicative of resource‐acquisitive trait strategies along the LES—may be able to maintain high rates of *V*_cmax_ and *J*_max_, even under elevated temperatures, through increased activity of Rubisco and other photosynthetic components in order to compensate for increased photorespiration due to climate warming (Tcherkez et al. 2006; Walker et al. 2016; Wingler et al. 1999).

In our study, Viognier, Cabernet sauvignon, and Cabernet franc had the highest rates of *V*_cmax_ and *J*_max_ on average (Figures 1 and 3), suggesting that these wine grape varieties might be more acclimated to increasing temperatures than the other varieties, such as Pinot noir and Riesling. Recent studies have also observed intraspecific variation in these physiological traits across a wide range of wine grape varieties (e.g., Gallo et al. 2021; Cui et al. 2023; Greer 2018). In addition, Gallo et al. (2021) found that Grenache grapevines were able to increase their photosynthetic capacity through enhanced *V*_cmax_ and *J*_max_ rates compared to Syrah in response to elevated temperatures. Therefore, high‐throughput techniques such as the DAT provide a promising approach for rapidly quantifying and predicting the responses of different wine grape varieties to warmer conditions in order to maintain a high rate of productivity in vineyards globally.

Due to the time constraints associated with steady‐state approaches of obtaining *A*‐*C*_i_ curves, it is challenging to measure physiological trait variation across the ~1,100 varieties of *Vitis vinifera* subsp. *vinifera* that are cultivated solely for wine production globally (Wolkovich et al. 2018). High‐throughput techniques such as the DAT are thus becoming increasingly important to rapidly and accurately evaluate the physiological basis for differences among varieties in response to climate change. The DAT can also be used to quantify trait variation and plant responses to environmental change in other economically important crop species (Tejera‐Nieves et al. 2024), particularly in regions that are more impacted by increasing temperatures.

## Conflicts of Interest

The authors declare no conflicts of interest.

## Data Availability

The data that support the findings of this study are openly available in the Borealis Repository—University of Toronto Dataverse at https://doi.org/10.5683/SP3/URPVFF.
